# The Impact of Sex on Cardiogenic Shock Outcomes Following ST Elevation Myocardial Infarction

**DOI:** 10.3390/jcm12196259

**Published:** 2023-09-28

**Authors:** Joshua H. Arnold, Leor Perl, Abid Assali, Pablo Codner, Gabriel Greenberg, Abid Samara, Avital Porter, Katia Orvin, Ran Kornowski, Hana Vaknin Assa

**Affiliations:** 1 Department of Medicine, University of Illinois at Chicago, Chicago, IL 60612, USA; 2 Department of Cardiology, Rabin Medical Center, Petach-Tikva 4941492, Israel; leorperl@gmail.com (L.P.);; 3 School of Medicine, Tel Aviv University, Tel-Aviv 6997801, Israel; 4 Department of Cardiology, Meir Medical Center, Kfar-Saba 4428164, Israel

**Keywords:** cardiogenic shock, STEMI, female

## Abstract

Background: Cardiogenic shock (CS) remains the leading cause of ST elevation myocardial infarction (STEMI)-related mortality. Contemporary studies have shown no sex-related differences in mortality. Methods: STEMI-CS patients undergoing primary percutaneous coronary intervention (PPCI) were included based on a dedicated prospective STEMI database. We compared sex-specific differences in CS characteristics at baseline, during hospitalization, and in subsequent clinical outcomes. Endpoints included all-cause mortality and major adverse cardiac events (MACE). Results: Of 3202 consecutive STEMI patients, 210 (6.5%) had CS, of which 63 (30.0%) were women. Women were older than men (73.2 vs. 65.5% y, *p* < 0.01), and more had hypertension (68.3 vs. 52.8%, *p* = 0.019) and diabetes (38.7 vs. 24.8%, *p* = 0.047). Fewer were smokers (13.3 vs. 41.2%, *p* < 0.01), had previous PCI (9.1 vs. 22.3% *p* = 0.016), or required IABP (35.3 vs. 51.1% *p* = 0.027). Women had higher rates of mortality (53.2 vs. 35.3% in-hospital, *p* = 0.01; 61.3 vs. 41.9% at 1 month, *p* = 0.01; and 73.8 vs. 52.6% at 3 years, *p* = 0.05) and MACE (60.6 vs. 41.6% in-hospital, *p* = 0.032; 66.1 vs. 45.6% at 1 month, *p* = 0.007; and 62.9 vs. 80.3% at 3 years, *p* = 0.015). After multivariate adjustment, female sex remained an independent factor for death (HR-2.42 [95% CI 1.014–5.033], *p* = 0.042) and MACE (HR-1.91 [95% CI 1.217–3.031], *p* = 0.01). Conclusions: CS complicating STEMI is associated with greater short- and long-term mortality and MACE in women. Sex-focused measures to improve diagnosis and treatment are mandatory for CS patients.

## 1. Introduction

Cardiogenic shock (CS) complicates 5% to 10% of cases of patients hospitalized with acute myocardial infarction (AMI) [1], and is the leading cause of death in this population [2,3,4]. Current guidelines suggest that primary percutaneous coronary intervention (PPCI) reduces mortality in these patients [5]. Thus, an early revascularization strategy has been adopted. However, despite current advances in reperfusion methods and the adoption of more aggressive strategies, CS continues to lead to significantly high and consistent mortality rates [6]. Early mortality rates have seen a decrease, but the 1-year survival rate has not changed [7].

In the setting of acute myocardial infarction, it has been repeatedly shown that differences in treatment remain between sexes [8,9,10], and that women presenting with AMI have higher adjusted early mortality rates [11]. Recent trends have shown increases in rates of hospitalization for women less than 65 years of age experiencing acute coronary syndrome (ACS) [12] and, when compared with men, women receive less medical and invasive therapy when suffering from AMI [10,13], including lower in-hospital use of coronary angiography [14] and PCI [15]. It has been suggested that the disparity may be due to higher baseline risk, atypical presentations, and underestimation of patient risk [14]. Although previous trials, including the SHOCK and SHOCK IABP-II trials, which evaluated the etiology, management, and outcomes of patients who developed CS following ST-elevation AMI (STEMI) have shown that mortality rates are high, no significant sex differences in mortality have been seen [16,17,18]. Identification of sex-related differences in outcomes for patients within this already very high-risk population can allow for changes in practice, such as earlier recognition and intervention, which may help mitigate these differences. The goal of this study was to assess any sex differences in clinical outcomes, including mortality, in patients presenting with acute STEMI complicated by CS and undergoing PPCI.

## 2. Materials and Methods

### 2.1. Patients and Setting

The clinical data of consecutive patients presenting with STEMI and treated by PPCI at the Department of Cardiology, Rabin Medical Center, Israel, between January 2009 and December 2018 were prospectively entered into a registry for purposes of monitoring patient-related variables and clinical events. Patients who were designated as Killip score 4 and with a primary clinical diagnosis of cardiogenic shock defined as having clinical and biochemical signs of hypoperfusion such as systolic blood pressure (SBP) <90 mmHg for ≥30 min, or required mechanical or medical support to maintain SBP ≥90 mmHg, end-organ hypoperfusion defined by urine output <30 mL/h, or cool extremities were identified for inclusion in the present post hoc study. Those who developed CS were compared between sexes. Exclusion criteria were treatment by thrombolysis and ineligibility for PPCI or a year-long dual antiplatelet regimen. The study was approved by the institutional review boards of the Rabin Medical Center.

### 2.2. Interventional Procedure

Patients provided explicit written informed consent before undergoing cardiac catheterization. In the event that patients were incapacitated due to mechanical ventilation, consent was obtained via the agreed decision of three physicians. Pretreatment consisted of aspirin and unfractionated heparin (70 U/kg), clopidogrel 300 or 600 mg, prasugrel 60 mg, or ticagrelor 180 mg, which was administered as a loading dose before or immediately after PCI. The utilization of glycoprotein IIb/IIIa inhibitors, and the choice of stent type and mechanical circulatory support devices, were left to the discretion of the primary operator. All stents were implanted with moderate to high deployment pressure (14 to 18 atm). All patients received dual antiplatelet therapy with aspirin 100 mg daily and a P2Y12 inhibitor (clopidogrel, prasugrel, or ticagrelor) for at least 12 months after PCI.

### 2.3. Study Endpoints

Immediate and in-hospital clinical events were prospectively recorded in the institutional database. During follow-up, patients completed standardized questionnaires on clinical events at 6-month intervals, either by telephone or in the outpatient clinic. When indicated, records from peripheral hospitals were acquired to verify the events. All events were further confirmed and adjudicated by the institutional clinical events adjudication committees. Survival status was assessed by the use of municipal civil registries at 1 and 3 years.

Clinical outcomes included all-cause mortality and major adverse cardiac events (MACE), which comprised death, myocardial infarction (MI), target vessel revascularization (TVR), and coronary artery bypass surgery (CABG). Other endpoints were peri-procedural arrhythmias, vascular complications, bleeding according to the thrombolysis in myocardial infarction (TIMI) classification, renal failure (defined as glomerular filtration rate below 50 mL/min/1.73 m^2^, calculated according to the Modification of Diet in Renal Disease formula), stent thrombosis, and cardiac death. Anemia was defined as hemoglobin levels lower than 13.0 g/dL for men and 12.0 g/dL for women. Findings were compared between male and female CS-STEMI patients.

### 2.4. Statistical Analysis

Continuous data are summarized as mean and standard deviation (SD) or median and interquartile range (IQR), and categorical data as frequency (%). Student’s *t*-test or analysis of variance was used to compare continuous variables between groups, and chi-square or Fisher’s exact test was used for categorical variables. The normality of variable distributions was assessed using the Kolmogorov–Smirnov test. Time-to-event curves were constructed using the Kaplan–Meier method and compared using the log-rank test. Cox regression was performed to determine independent predictors of the primary endpoint, accounting for known baseline cardiovascular risk differences. Effect sizes are presented as hazard ratio (HR) and 95% confidence interval (CI). Stepwise variable selection of significant univariate predictors (*p* < 0.1) was used to identify variables for inclusion in the multivariate model. All statistical analyses were performed with IBM SPSS V.28 (IBM Corp. Armonk, NY, USA). A *p* value of <0.05 was considered statistically significant.

## 3. Results

Among the 3202 consecutive STEMI patients included in our analysis, 210 (6.5%) suffered from CS. Of these, 147 (70.0%) were men and 63 (30.0%) were women. Women were older than men (73.2 ± 12.4 vs. 65.5 ± 12.0 years, *p* < 0.01) and had higher BMIs (36.7 ± 10.9 vs. 31.3 ± 10.3 *p* = 0.002). Among comorbid conditions, women were less frequently smokers (31.1 vs. 40.4%, *p* = 0.006), and less likely to have previously undergone PCI (9.1 vs. 22.3% *p* = 0.016). Women more frequently had hypertension (68.3 vs. 52.8%, *p* = 0.019) and diabetes mellitus (38.7 vs. 24.8%, *p* = 0.047). There were no other differences found in baseline characteristics between the two sexes (Table 1). Ischemic time (5.1 vs. 4.1 h, *p* = 0.528) and door to balloon time (1.7 vs. 1.5 h, *p* = 0.525) did not differ between the sexes, nor did the use of IV inotropes or the use of a temporary pacemaker. Women were less likely to receive intra-aortic balloon pump (IABP) treatment (35.3 vs. 51.1% *p* = 0.027) compared with men. Target vessels were smaller in female patients, as represented by the use of an average stent diameter of 3.0 vs. 3.2 (*p* = 0.028) in male patients. Men were more often treated with multivessel angioplasties as compared with women (2.3 vs. 2.1 vessels, *p* = 0.077), but their SYNTAX scores did not differ. Adherence to antiplatelet therapy was equal in both sexes (Table 2).

**Figure 1:**
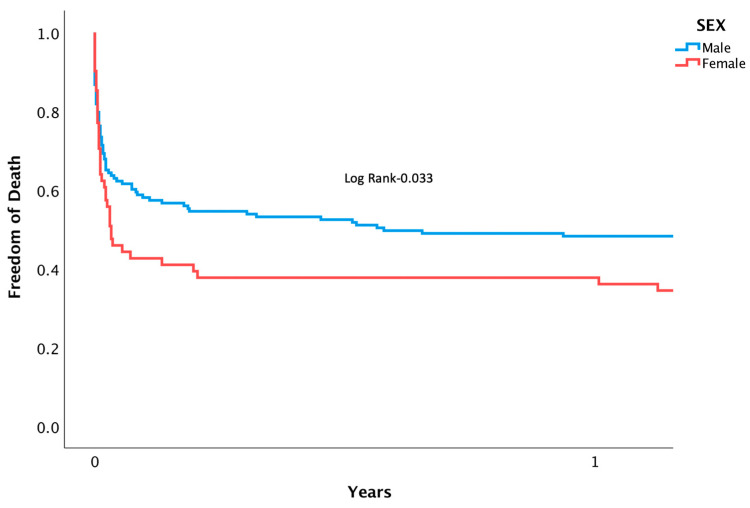
Kaplan-meier curve displaying length of time patients were free from death following PPCI for AMI complicated by CS.

**Figure 2:**
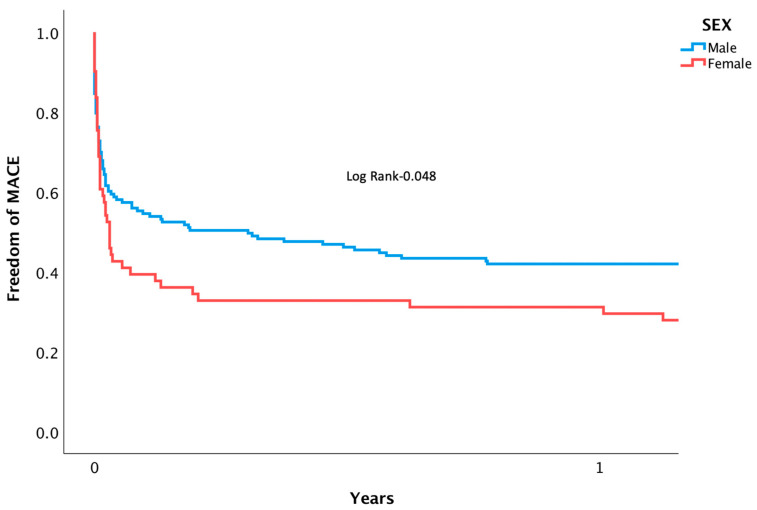
Kaplan-meier curve displaying length of time patients were free from MACE following PPCI for AMI complicated by CS.

Patients suffering from CS who underwent PPCI were followed for rates of mortality and MACE. Mortality at separate time points was found to be significantly higher among women during in-hospital (53.2 vs. 35.3%, *p* = 0.01), 1-month (61.3 vs. 41.9%, *p* = 0.01), and after 3 years (73.8 vs. 52.6%, *p* = 0.05) following intervention (Figure 1). A Cox regression analysis was conducted, adjusting for confounding variables, and mortality remained higher for women (HR 2.42 [95% CI 1.014–5.033], *p* = 0.042). Other factors associated with increased risk of mortality included: diabetes mellitus (HR 2.81 [95% CI 1.055–4.345], *p* = 0.038), renal failure (HR 3.08 [95% CI 1.201–6.038], *p* = 0.02), and PVD (HR 4.23 [95% CI 1.640–7.496], *p* = 0.003). Notably, age, the usage of an IABP, the timing of day versus night, and door-to-balloon time were not found to have an impact on rates of mortality (Table 3). Rates of MACE were higher among women during their admission (60.6 vs. 41.6%, *p* = 0.032), at 1-month (66.1 vs. 45.6%, *p* = 0.007), and at 3 years (62.9 vs. 80.3, *p* = 0.015) (Figure 2). (For the individual components, please see Supplementary Table S1). After multivariate analysis, MACE rates remained higher for women (HR 1.91 [95% CI 1.217–3.031], *p* = 0.01). Other factors associated with increased risk of MACE included age (HR 1.12 [95% CI 1.126–1.324], *p* = 0.005), renal failure (HR 1.88 [95% CI 1.189–2.823], *p* = 0.007), and PVD (HR 1.83 [95% CI 1.072–2.823], *p* = 0.021) (Table 4). Rates of recurrent myocardial infarction, stent thrombosis, and the need for revascularization did not differ between sexes at all time points.

## 4. Discussion

In this study examining the course of STEMI patients treated with PPCI presenting with CS, we observed that female patients suffer from worse short- and long-term outcomes when compared with men. Information regarding the worse outcomes experienced by women suffering from CAD and ACS requiring PCI is well known [10,11,12,19,20,21]. However, outcomes for women compared with men in CS following STEMI have yet to be clearly defined. In this observational study, we compared mortality rates of women and men within this population with the intention of further clarifying sex differences in clinical outcomes and management. Considering the already poor outcomes for patients suffering from CS following STEMI, recognition of differences between the sexes is necessary.

Previous studies have suggested that sex differences can be attributed to the fact that women present at older age or with an increased burden of comorbidities and that, when these differences in baseline characteristics are controlled for, outcome differences do not exist [22,23]. Other studies looking specifically at CS patients undergoing PPCI following AMI, including the SHOCK registry, also showed no differences in outcomes between the sexes [24,25], or that sex is not independently associated with clinical outcomes in similar populations [26]. However, other studies have found that women are at increased risk for in-hospital mortality [27,28] and that, in general, women are more likely to die in hospital following AMI [29]. Women similarly show higher unadjusted mortality rates 24 h after randomization, as published in the IABP-II sub study trial (18% vs. 9%, *p* = 0.004) [30]. A decrease in cardiac power, a measure from the SHOCK trial defined as the product of cardiac output and mean arterial blood pressure, was found to be the strongest independent correlate with in-hospital mortality, and was significantly associated with female sex [31]. However, the inconsistency in study results is an example of the discordance in information regarding sex and mortality outcomes within this population.

Our study found higher rates of death in women at three separate time points: during admission, after 1 month, and after 3 years. In our multivariate model, PVD was the strongest predictor of mortality from CS. Increased in-hospital CV morbidity and mortality in patients with PVD and ACS has been reported [32,33]. This has been thought to be related to an increased systemic atherosclerotic burden, impaired arterial remodeling, and increased disease progression [34,35], all of which may contribute to the development of CS and the deterioration of patients in an acute setting. Of important note, this study also assessed other possibly contributing elements, including clinical factors such as previous MI, left ventricular function, and the timing of the procedure, namely door-to-balloon and ischemic time. We also assessed procedural characteristics, including use of a radial versus femoral approach and non-culprit vessel angioplasty. None of these variables showed statistical significance in relation to mortality, contradictory to previous studies [36,37,38,39]. The contrasting results may be due in part to variability in the definitions of shock parameters and measurements across studies [36]. Another explanation may be that the time patients spent in shock was an important risk factor, but this data was not collected.

Female patients were more likely to be older and to be diagnosed with diabetes mellitus and hypertension. However, after multivariate analysis, a significant difference in mortality between the sexes remained. Women tend to delay health care-seeking behavior [40], and biological sex differences in the extent of endothelial dysfunction, an important prognostic factor for patients with AMI [41], exist. These differences have been postulated to be due to higher rates of inflammation, loss of arterial compliance, and increase in remodeling possibly due to loss of sex-protecting hormones [42]. Estrogen has shown a similar role in premenopausal women in reducing inflammation in the setting of systemic inflammatory response syndrome (SIRS) complicated by increases in inflammatory cytokines and mediators from systemic hypoperfusion [43]. Complicating the matter further is the fact that women have a higher prevalence of CS following STEMI, compared with men [27,43,44,45], consistent with our cohort (10.9% vs. 5.7%). This could be partly attributed to the fact that women are at increased risk for more sex-specific triggers of CS, such as peripartum cardiomyopathy, microvascular dysfunction, spontaneous coronary artery dissection (SCAD), and takotsubo cardiomyopathy [46].

It has been well-established that attempts at all but timely PPCI in the treatment of CS complicated by STEMI fail to prevent short- and long-term MACE [47,48,49]. As discussed, although rates of in-hospital mortality have decreased over time, CS remains the most common cause of death. Increased use of angiography, the adoption of an earlier revascularization strategy, and the increased rates of administration of novel P2Y12 inhibitors and statins may be contributing to the relative decline in death [50,51,52]. However, even with the increased use of early revascularization, mortality rates remain high, and differences in outcomes exist between men and women [2]. It has been suggested that mortality in STEMI complicated by CS may be reduced by emphasizing rapid LV unloading and immediate coronary revascularization [53], which require even more prompt initiation of treatment, especially in women. Our study’s patient population was similar to the one found in a recent study by Yahn et al., which also analyzed sex-differences in outcomes for CS patients [54]. This larger single center study found no differences in outcomes between sexes; however, it did note a significant disparity in utilization of mechanical circulatory support (MCS). Our study also revealed women to be less likely to be treated with MCS, but showed no difference in use of IV inotropes. This may account for differences in outcomes within our smaller patient cohort; however, no study, as of yet, has been able to show a significant improvement in rates of mortality for patients with CS-AMI who are treated with MCS devices such as IABP or impella, which are used with the aim to aid in rapidly unloading the left ventricle and improving coronary blood flow [17,55]. Reasons for women to be less likely to receive MCS may be related to under-recognition of the severity of CS in this population. Large, randomized control trials investigating the use of newer MCS devices, such as impella, in the setting of improved treatment measures previously mentioned are necessary. Furthermore, implementing and further validating a cardiogenic shock classification system, such as the SCAI classification, may also improve outcomes [56].

## 5. Study Limitations

This study is an observational post hoc analysis of our cohort, a design that in itself presents limitations. It is also a single-center trial. Although based on an all-comer prospective registry, biases may have influenced the selection of data, the method of therapy, and other factors associated with outcome, although our main discussion topic remained significant after multivariate analysis. The observational nature of the study also prevents us from attributing a causal relationship between female sex and mortality. Finally, as rates of CS are relatively low, our final analysis included a relatively small number of patients. Nevertheless, this is one of the largest real-world studies comparing outcomes in patients suffering from CS complicating STEMI, and it shows a clear independent impact of female sex on worse prognosis.

## 6. Conclusions

Our study revealed a significant increase in mortality in women compared with men suffering from cardiogenic shock after STEMI and undergoing PPCI. The recognition of the increased risk is necessary to better steer management and improve prognosis for female patients. We anticipate that future studies assessing differences in outcomes will shed more light on this important topic and enable better care of both male and female patients suffering from this ominous medical condition.

## Figures and Tables

**Table 1:** Baseline characteristics.

Characteristics	Men (*n* = 147)	Women (*n* = 63)	*p* Value
Age (years)	65.5 ± 12.0	73.2 ± 12.4	<0.001
BMI (kg/m^2^)	31.3 ± 10.3	36.7 ± 10.9	0.002
Diabetes mellitus type 2 (%)	24.8	38.7	0.047
Family history of CAD (%)	19.0	20.4	0.838
Hypertension (%)	52.8	68.3	0.019
Hyperlipidemia (%)	49.6	54.1	0.566
Previous PCI (%)	22.3	9.1	0.016
Previous MI (%)	20.9	13.0	0.126
Obesity (%)	27.8	35.8	0.285
Smoking (%)	40.4	31.1	0.006
S/P CABG (%)	7.9	7.5	0.941
CVA (%)	11.0	13.0	0.711
PVD (%)	10.2	17.3	0.193
Renal failure (%)	31.0	40.4	0.217

Abbreviations: CAD, coronary artery disease; PCI, percutaneous coronary intervention; MI, myocardial infarction; CABG, coronary artery bypass surgery; CVA, cerebrovascular accident; PVD, peripheral vascular disease.

**Table 2:** Procedural characteristics.

	Men (*n* = 147)	Women (*n* = 63)	*p* Value
MI Location			0.340
Anterior (%)	43.5	45.2	
Inferior (%)	41.3	39.9	
Average number of diseased vessels (n)	2.3	2.1	0.077
Mean SYNTAX Score	16.8 ± 17.7	16.9 ± 13.8	0.818
Thrombectomy (%)	21.3	14.5	0.262
TIMI flow grade prior to intervention	0.8	0.7	0.465
TIMI flow grade after intervention	2.9	2.8	0.215
Stent placement (%)	94.9	90.3	0.235
Average stent diameter (mm)	3.2	3.00	0.028
Average stent length (mm)	20.2	21.0	0.429
Successful PCI (%)	89.7	87.1	0.590
Aspirin (%)	76.0	81.4	0.469
Clopidogrel (%)	33.9	42.9	0.298
Prasugrel (%)	30.0	11.1	0.288
Symptoms to FMC (hours)	5.1	4.1	0.528
FMC to balloon (hours)	1.7	1.5	0.525
Nighttime procedure (%)	47.1	46.8	0.970
IABP (%)	51.1	35.3	0.027
LVEF % at presentation	32.3	31.0	0.377
Use of inotropes (%)	63.1	61.7	0.866
Temporary pacemaker (%)	30.7	42.3	0.139

Abbreviations: TIMI, thrombolysis in myocardial infarction; PCI, percutaneous coronary intervention; FMC, first medical contact; IABP, intra-aortic balloon pump; LVEF, left ventricular ejection fraction.

**Table 3:** Cox analysis for mortality.

	HR	Range	*p* Value
Age	1.03	0.992–1.078	0.104
Sex	2.42	1.014–5.033	0.042
Diabetes mellitus type 2	2.81	1.055–4.345	0.038
Hypertension	0.54	0.223–1.458	0.253
Family history	1.14	0.297–3.475	0.801
Renal failure	3.08	1.201–6.038	0.020
CVA	1.78	0.634–3.207	0.429
PVD	4.23	1.640–7.496	0.003
Previous PCI	0.25	0.224–1.642	0.237
S/P CABG	1.20	0.285–5.374	0.822
MI Location (Anterior)	1.12	0.944–1.356	0.174

Abbreviations: CVA, cerebrovascular accident; PVD, peripheral vascular disease; PCI, percutaneous coronary intervention; MI, myocardial infarction; CABG, coronary artery bypass surgery.

**Table 4:** Cox analysis for MACE.

	HR	Range	*p* Value
Age	1.12	1.126–1.324	0.005
Sex	1.91	1.217–3.031	0.010
Diabetes mellitus type 2	1.51	1.039–2.428	0.035
Hypertension	1.23	0.833–2.026	0.283
Family history	0.92	0.536–2.035	0.321
Renal failure	1.88	1.189–2.823	0.007
CVA	1.09	0.682–1.923	0.703
PVD	1.83	1.072–2.823	0.021
Previous PCI	0.80	0.539–1.501	0.438
S/P CABG	1.80	0.878–2.733	0.182
MI location (anterior)	1.22	0.553–2.104	0.801

Abbreviations: CVA, cerebrovascular accident; PVD, peripheral vascular disease; PCI, percutaneous coronary intervention; CABG, coronary artery bypass surgery; MI, myocardial infarction.

## Data Availability

Not applicable.

## Supplementary Materials

The following supporting information can be downloaded at: https://www.mdpi.com/article/10.3390/jcm12196259/s1, Table S1: Outcomes.
