# Supplementary material for: The Impact of Sex on Cardiogenic Shock Outcomes Following ST Elevation Myocardial Infarction
Source: J Clin Med. 2023 Sep 28;12(19):6259. doi: 10.3390/jcm12196259 (PMC10573491; doi:10.3390/jcm12196259)
Supplement: Supplementary file 1 [file jcm-12-06259-s001.zip › jcm-2593766-supplementary.pdf]

Supplementary Table S1. Outcomes

	Men (n = 147)	Women (n = 63)	*p* value
**In hospital**			
MI	3.0	4.9	0.497
Cardiac arrest	20.1	19.0	0.851
Arrhythmia	12.9	11.3	0.770
Vascular	2.3	0.0	0.246
Tamponade	1.5	1.6	0.934
Stent thrombosis	2.2	0.0	0.241
TVR	1.5	1.6	0.940
CVA	0.1	1.6	0.183
Renal failure	25.9	34.4	0.225
CABG	0.0	0.0	-
MACE	41.6	60.6	0.032
Death	35.3	53.2	0.017
**1 month**			
MI	5.1	6.5	0.712
AST	2.2	1.6	0.785
TVR	2.9	0.0	0.174
TLR	2.2	0.0	0.174
CVA	3.7	1.6	0.435
Renal failure	4.5	6.5	0.570
LVEF %	32.2	30.4	0.566
CABG	0.0	0.0	-
MACE	45.6	66.1	0.007
Death	41.9	61.3	0.011
**1 year**			
MI	7.4	11.3	0.362
TVR	8.1	3.2	0.202
TLR	7.4	3.2	0.261
ISR	6.6	4.8	0.629
Stent thrombosis	2.2	1.6	0.785
CABG	2.2	1.6	0.785
MACE	56.6	72.6	0.032
Death	50.0	66.1	0.034

Abbreviations: MI, myocardial infarction; TVR, target vessel revascularization; CVA, cerebrovascular accident; CABG, coronary artery bypass surgery; MACE, major adverse cardiovascular events; AST, acute stent thrombosis; TLR, target lesion revascularization; LVEF, left ventricular ejection fraction; ISR, in-stent restenosis.
